# Assessment of the Film-Free Water Decal Method for Speckle Pattern Application in Digital Image Correlation

**DOI:** 10.3390/s24175657

**Published:** 2024-08-30

**Authors:** Anna Camille Sanchez, Dong-Keon Kim

**Affiliations:** 1 Department of ICT Integrated Ocean Smart Cities Engineering, Dong-A University, Busan 49304, Republic of Korea; acsanchez.ce@gmail.com; 2 National Core Research Center for Disaster-Free and Safe Ocean Cities Construction, Dong-A University, Busan 49304, Republic of Korea

**Keywords:** film-free water decal, digital image correlation (DIC), speckle pattern, SS275 steel, strain measurement, tensile testing, speckle pattern application

## Abstract

Digital Image Correlation (DIC) often encounters challenges with variability and consistency in traditional speckle pattern application techniques, such as spray-painting, affecting measurement accuracy and reliability. This study evaluates a film-free water decal method as an alternative for applying speckle patterns in DIC. SS275 structural steel specimens were prepared with speckle patterns using both the film-free water decal method and traditional spray-painting. The quality of the speckle patterns was assessed, and their effectiveness for DIC was evaluated through tensile testing and a comparison with strain gauge measurements. The film-free water decal method provided enhanced control over speckle pattern application, resulting in high-quality, consistent patterns. Strain measurements obtained using this method closely matched those from traditional methods, confirming its reliability. The film-free water decal method offers a practical and reliable alternative to spray-painting, improving the consistency and accuracy of DIC experiments, with potential applications in various engineering and scientific fields.

## 1. Introduction

Digital Image Correlation (DIC) is a widely utilized non-contact optical technique for measuring full-field displacement and strain on the surface of specimens under load [1,2,3]. Its applications extend across material science [4,5,6], structural engineering [7], and biomechanics [8], owing to its capacity to deliver precise and detailed deformation and strain distributions. Recent studies have demonstrated the versatility of DIC in various unconventional applications, including the material characterization of fish skin [9], facial strain analysis [10], and non-invasive vibrational analysis of mechanical systems [11]. Additionally, a systematic literature review on DIC applications in laboratory structural tests highlighted their effectiveness across a wide range of materials and test types, including concrete, steel, composites, and masonry [12]. These studies underscore the adaptability of DIC beyond traditional engineering applications, showcasing its potential in diverse fields.

The accuracy of DIC measurements relies heavily on the quality and consistency of the speckle pattern applied to the specimen’s surface. Speckle patterns are indispensable in DIC experiments, providing the necessary texture for tracking surface displacements and deformations [13]. High-quality speckle patterns must exhibit high contrast, randomness, appropriate size, and stability to ensure accurate and reliable DIC analysis [14,15]. Poor-quality speckle patterns can lead to significant errors in displacement and strain measurements, compromising the integrity of the experimental data [11]. Because of their critical role in DIC, there are many studies related to speckle patterns, including the development of assessment metrics for pattern quality [16,17,18], digital design and optimization techniques of speckle patterns [19,20,21], application methods for speckle patterns on various material surfaces and scales [15], and innovations, such as employing color speckle patterns with a prism camera to improve measurement accuracy in complex deformation scenarios [22].

In DIC, each speckle is represented by a distinct gray level in the digital image, resulting from varying light intensities reflected from the surface. The intensity of each pixel is recorded as a gray level, typically ranging from 0 (black) to 255 (white) in an 8 bit grayscale image [13]. The distribution and contrast of these gray levels are crucial for accurate correlations and significantly influence the accuracy and reliability of DIC measurements.

The DIC process involves dividing the image into smaller regions called subsets, each containing a unique speckle pattern. As the specimen deforms, these subsets move, and their movements are tracked using correlation algorithms. By matching the speckle patterns before and after deformation, displacement fields are calculated, which are then used to derive strain and other deformation metrics, providing comprehensive data on the material’s behavior [1,23].

Traditional speckle pattern application techniques, such as spray-painting, often face challenges related to variability in and consistency of pattern quality. Inconsistencies in speckle size, density, and adhesion can lead to significant errors in DIC measurements, affecting the accuracy and reliability of the results [15,24]. Previous research has explored alternative methods to overcome these issues [15], including lithography [25], spin-coating [26], focused ion beam [27], and high-temperature air-plasma applications [28].

Chen observed that a common approach in previous research was to create digital speckle patterns (DSPs) using a computer and then transfer these generated patterns onto specimens for analyzing both large-scale and small-scale deformations [21]. This transfer printing method offers a significant advantage, particularly for metals, as it does not alter the mechanical properties of the specimen. Given these benefits, various studies have focused on using special papers for applying DSPs on metal surfaces. However, each of these methods also presents its own set of limitations, such as the need for specialized equipment, complex procedures, or suitability constraints depending on the material and conditions. 

Studies by Gualtieri and Mazzoleni [29,30] employed a thermochemical process with high-temperature ironing, which is unsuitable for materials that cannot withstand high temperatures, and is limited in effectiveness on painted surfaces. Additionally, their method often required significant surface preparation, such as sandblasting, to achieve sufficient surface roughness for better adhesion. Chen and Quino [31,32] utilized multiple layers of transfer paper and specialized printing methods, requiring complex assembly and extensive preparation, making them less practical for diverse experimental settings. Despite these advancements, the need remains for a simpler, more effective method that can be easily adopted in various experimental settings without extensive preparation, specialized equipment, or additional surface treatments.

The film-free water decal method introduces a novel approach to speckle pattern application, offering potential improvements in consistency and quality. This method utilizes commercially available water decal paper to apply high-contrast, high-adhesion speckle patterns, aiming to address the limitations of traditional spray-painting [33]. The significance of this study lies in its potential to enhance the reliability and practicality of DIC by providing a more controlled and uniform speckle pattern application method.

This study aims to comprehensively evaluate the quality and suitability of speckle patterns produced by the film-free water decal method compared to traditional spray-painting techniques. Given that DIC is still in its early stages of development and standardized accuracy tests for these methods are not yet established, this study adopts a comparative approach to address several key objectives.

Firstly, it seeks to evaluate the quality, adhesion, and durability of speckle patterns applied using the film-free water decal method relative to traditional spray-painting. Secondly, the study aims to assess the accuracy and reliability of deformation measurements, including strain and displacement, obtained using the film-free method in comparison to traditional methods. Thirdly, the study intends to validate the material property measurements derived from DIC using the film-free method against those obtained from Universal Testing Machines (UTMs). By addressing these objectives, the study aims to establish the film-free water decal method as a viable alternative for speckle pattern application in DIC, offering significant improvements in pattern quality and application consistency.

The structure of this paper is designed to systematically address the research objectives and provide a comprehensive analysis of the proposed film-free water decal method for DIC. Section 2 provides a thorough review of the existing transfer printing methods for the speckle pattern application on metals, focusing on both thermo-mechanical (Section 2.1) and water-based techniques (Section 2.2), and highlighting the unique challenges associated with each of them. This review sets the context for the development of the film-free method. Section 3 details the materials and methods used in this study. It begins with the principles underlying the film-free water decal method (Section 3.1.1), and then discusses the development and optimization processes (Section 3.1.2) to achieve optimal speckle pattern applications. This section also outlines the transfer procedure for applying the film-free decal to metal specimens (Section 3.1.3), including demonstrations of successful applications for various point sizes. Section 3.2 focuses on the experimental validation of the proposed method, detailing the experiment design (Section 3.2.1), specimen preparation (Section 3.2.2), experimental setup (Section 3.2.3), and DIC setup and processing (Section 3.2.4). Section 4 presents the results of the study, covering the quality assessment of the speckle patterns (Section 4.1), validation of strain measurements (Section 4.2), and evaluation of material properties (Section 4.3). Finally, Section 5 concludes the paper, summarizing the key findings and implications of the study.

## 2. Review of Existing Transfer Printing Methods for Speckle Pattern Application on Metals

### 2.1. Thermo-Mechanical Transfer Methods

In 2012, Gualtieri developed a method to apply speckle patterns onto metal surfaces using transfer printing [29]. His approach involved generating speckle patterns on a computer, printing them onto various types of paper, and then transferring these patterns onto specimens by ironing. The primary goal was to transfer ink from the printer to the paper and subsequently from the paper to the specimen.

Gualtieri [29] tested three types of paper—standard printing paper, photographic paper, and a plastic sheet—and found the plastic sheet to be the most effective, as it allowed the ink to be deposited on the paper without being absorbed, facilitating later transfer to the specimen. During the process, he observed discrepancies in the sizes of the speckle dots, noting variations between the designed, printed, and ironed patterns. This discrepancy in pattern size undermines the optimization and design of speckle properties.

Additionally, the ironing process proved to be sensitive to time, temperature, and pressure; a slight deviation in any of these parameters could result in incomplete transfer or melting of the plastic sheet. Importantly, these values are dependent on the material type and dimensions. The sample also needed to be immersed in water to remove the plastic sheet, making the process messy and time-consuming.

For optimal results, Gualtieri [29] emphasized that the surface should be as flat as possible because the plastic sheet cannot follow the crest of an uneven sample piece. His method was not effective on painted white surfaces, as the pattern did not adhere well to the paint. The solution he found to be most effective for surface preparation was sandblasting, which provided a matte finish and sufficient contrast with black speckles.

Gualtieri’s study [29] utilized accessible materials such as a plastic sheet, laser printer, and electric iron to apply speckle patterns on metal surfaces. While effective, the method posed challenges, including sensitivity to ironing time, temperature, pressure, and the need for uniform heat distribution. Issues with pattern size discrepancies, limitations on sample size, and difficulties with painted surfaces were also significant. Additionally, the method required extensive surface preparation, which can be complex and time-consuming.

Mazzoleni later developed a method similar to Gualtieri’s, which he termed “thermo-mechanical toner transfer” [30] in 2015. This technique also involves transferring melted toner from printed paper to the specimen surface using a flat iron, but with one key difference: Mazzoleni used glossy photo paper suitable for inkjet printers instead of a plastic sheet. This choice introduced its own set of challenges.

Mazzoleni [30] encountered similar issues to those observed by Gualtieri, particularly discrepancies between the designed speckle pattern dot sizes and those transferred onto the specimen. His study required tuning the speckle sizes to achieve a diameter as close as possible to the intended design. He attributed these discrepancies to toner smudging during the transfer process. However, this issue may also stem from a material mismatch, as photo papers are designed for inkjet printers and may not be suitable for use with toner ink from a laser printer. The coating on photo paper is engineered to work with inkjet printer ink, and using it in a laser printer can lead to problems such as smudging, poor print quality, and even potential damage to the printer due to the paper’s inability to handle the laser printer’s heat.

Although Mazzoleni [30] was able to apply his method to spray-painted white 5 mm thick aluminum samples, this approach raises concerns because paint can be sensitive to extreme heat, depending on the specific pigments and additives used. Furthermore, the effectiveness of his method on painted surfaces remains uncertain, as his experiments were primarily conducted on sandblasted specimens rather than painted ones. The authors acknowledged a key limitation of the toner transfer technique, noting that it is suitable only for surfaces that can withstand heating to around 100 °C for a few minutes without incurring physical damage or altering their mechanical properties.

### 2.2. Water-Based Transfer Methods

**Figure 1:**
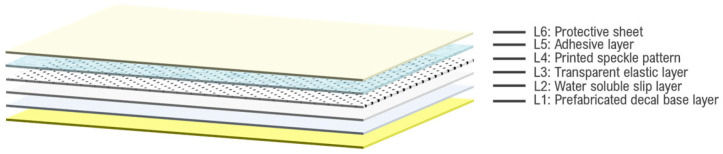
Schematic representation of the six-layer structure used in Chen’s Water Transfer Printing method for applying digital speckle patterns.

In the same year, Chen et al. developed a method for transferring digital speckle patterns using the Water Transfer Printing (WTP) technique [31]. The transfer paper used in this method consists of six layers, grouped into three main parts: the prefabricated decal part, the printed speckle pattern part, and the protective part. The base layer (L1) is an absorptive paper covered with a water-soluble layer (L2), both prefabricated in a factory. On top of this, a transparent elastic layer (L3) is applied through solid printing, coating, or screen printing. The designed speckle pattern is then printed twice on this elastic layer, forming layer L4. An adhesive layer (L5) is added next, followed by a protective sheet (L6) to prevent contamination. The key principle of this method is that the water-soluble slip layer dissolves during the process, leaving the printed speckle pattern on the specimen’s surface. Figure 1 provides a schematic representation of the six-layer structure used in this method.

To transfer the speckle patterns onto the specimen, the surface is first cleaned with acetone or alcohol and coated with matte white paint. The protective sheet layer is then removed, and the adhesive layer is placed face down on the specimen, with the prefabricated decal facing up. The prefabricated decal is then evenly moistened using a brush or cloth to activate the adhesive layer, which binds the printed layer and transparent elastic layer to the specimen’s surface. Once the decal is uniformly damp, it is gently removed, allowing the water-soluble slip layer to dissolve and leave the adhesive, printed, and transparent elastic layers on the specimen’s surface. The specimen is then left to dry at room temperature, after which it is ready for the DIC experiment.

Chen and his colleagues [31] demonstrated the effectiveness of their method through experiments. However, the process of creating the “portable transfer paper” is complex and involves materials that are not as easily accessible as those used in other methods. The base layer, for example, is prefabricated in a factory. Additionally, assembling the transfer paper requires specialized printing techniques, such as screen printing, gravure, and lithography—processes typically found in industrial or commercial settings. Unlike the methods developed by Gualtieri [29] and Mazzoleni [30], which rely on a laser printer and special paper, Chen’s approach demands specialized skills in printing and the assembly of multiple layers. This complexity makes the method less straightforward and not as easily accomplished with common household or office items.

**Figure 2:**
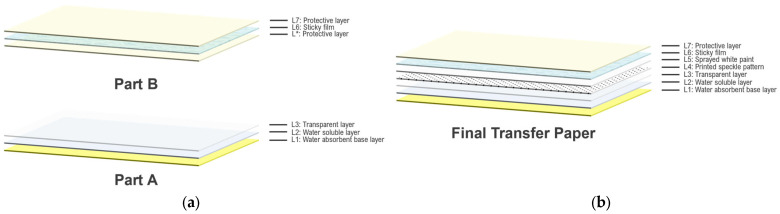
Schematic representation of the “tattoo speckle pattern” system used by Quino et al.: (**a**) Part A and Part B components, each with their respective layers; (**b**) final assembled transfer paper ready for use.

In 2020, Quino and his team developed a method similar to Chen’s, which they referred to as the “tattoo speckle pattern” technique [32]. This approach utilized a commercially available A4-sized laser tattoo set to transfer a custom-designed speckle pattern onto the specimen. The system consists of two main parts, as illustrated in Figure 2, with a total of seven layers.

The procedure begins by printing the digital speckle pattern onto layer L3 of Part A, thereby creating layer L4. A coating of white paint is then sprayed onto layer L4 and allowed to dry, forming layer L5. The protective layer, L*, in Part B of the tattoo system is removed next, exposing the adhesive film layer, L6, which is carefully affixed to the previously treated L5 layer of Part A, combining the two parts. Quino and his team [32] recommend using a rigid ruler during this process to ensure a seamless application and minimize the formation of air pockets. Once the two parts are combined, the resulting speckle system is ready for use.

To apply the speckle pattern to the specimen, the necessary portion of the speckle system is cut from the sheet, the protective layer, L7, is peeled off, and the exposed adhesive side is placed onto the specimen. The specimen is then moistened, allowing the water-soluble layer, L1, to dissolve and the speckle pattern to adhere to the surface.

Quino and colleagues [32] utilized a commercially available tattoo set, designed for either inkjet or laser printers, to transfer custom-designed speckle patterns onto specimens. The principle of transfer is similar to Chen’s method; however, the assembly of the tattoo paper system involves fewer steps, and only a laser printer is required to print the speckles. Unlike Chen’s method, Quino’s approach includes the additional step of spraying white paint onto the transfer paper, adding another layer to their system. Although the tattoo set is commercially available, the proper assembly of the two parts remains critical. The adherence of the speckle pattern to the specimen depends on the integrity of the sticky film layer and the accurate placement of the other layers on this film.

While Quino’s method [32] claims to reduce specimen preparation time due to the pre-existing white background on the paper, the need to apply white paint and allow it to dry is an integral part of creating the tattoo paper. This process does not necessarily shorten the overall time; rather, it shifts the time requirement to another step. Although preparing the speckle system in advance offers some convenience, it is important to consider the ductility of the paint, as recommended by iDICs guidelines [14]. For ductile materials, the paint should exhibit maximum ductility to ensure it stretches with the underlying test piece without cracking or debonding. Therefore, tests should be conducted shortly after painting to maintain optimal ductility. Prolonging the time before using the tattoo paper may result in the paint fully curing, becoming brittle, and less effective.

Both Chen’s [31] and Quino’s [32] methods place the printed digital speckle patterns in the middle layer of the transfer paper. This setup requires the entire layering process to be repeated each time a different digital speckle pattern (with varying parameters, such as size and density) is needed. After applying the transfer paper, the layers that remain on the surface include the adhesive layer, the printed layer, and the transparent elastic layer. This structure is similar to traditional water decals, which leave films on the surface. These films can be made from various materials and vary in thickness. While they protect the printed layer, their characteristics and chemical composition can impact the durability, flexibility, and adhesion of the speckle pattern onto the specimen. A common issue with these films is peeling; if a small area cracks, the peeling often spreads across the entire surface. This problem is particularly significant in large strain experiments, where maintaining the integrity of the film is crucial.

Upon reviewing the methodologies and challenges presented in Gualtieri’s [29], Mazzoleni’s [30], Chen’s [31], and Quino’s [32] studies, it becomes evident that each approach introduces unique complexities and limitations to the application of speckle patterns for DIC analysis. The intricate processes involved, from time-sensitive ironing to the complex assembly of transfer papers, not only demand attention, but also raise questions about the efficiency and practicality of existing methods. While these studies contribute valuable insights into the field, they underscore the need for a more optimal, user-friendly, and universally applicable speckle pattern transfer technique—one that enhances accuracy, repeatability, and ease of implementation in experimental mechanics.

In response to the challenges presented by existing methods, the film-free method is introduced as a simpler and more effective approach for transferring digital speckle patterns onto metals. This method eliminates the need for extensive preparation, specialized equipment, or additional surface treatments, making it accessible for a wide range of experimental settings. The film-free method utilizes a commercial water decal that can be easily applied by printing the digital speckle patterns using a standard laser printer, followed by a straightforward transfer process. The process involves both heat, applied through blow drying, and water, used to wet the paper, ensuring a reliable and efficient transfer of the speckle pattern onto the specimen. This approach combines the benefits of existing techniques while simplifying the application process, offering a practical solution for DIC experiments.

## 3. Materials and Methods

### 3.1. Film-Free Water Decal Method

#### 3.1.1. Principle

The water decal used in this study was a commercially available product from Sunnyscopa. Specifically, the chosen product was the Film-Free Waterslide Decal Paper Type A, which is specifically designed for ceramics, glasses, and coated steels. This type of decal paper can be printed using a standard laser printer and leaves only toner ink after transfer.

**Figure 3:**
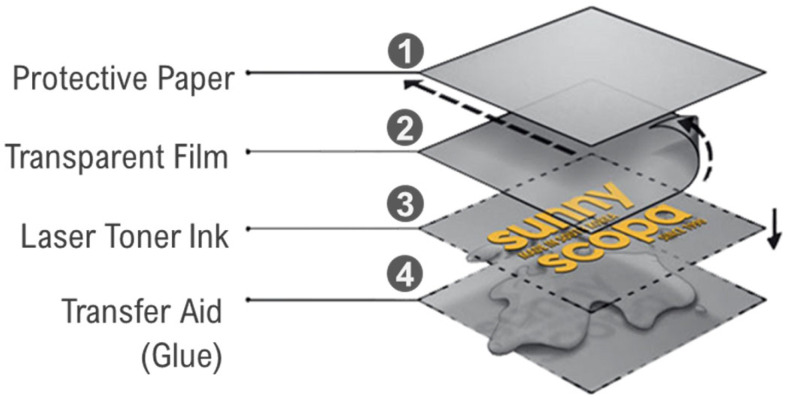
Structure and mechanism of Sunnyscopa film-free water decal paper: toner ink is printed on the transparent film, applied to the specimen with glue, and the protective paper and transparent film are removed, leaving the toner ink on the surface.

According to Sunnyscopa, the Film-Free Waterslide Decal Paper is composed of four layers (Figure 3) [34]:

1. Protective paper: Protects the underlying layers.
2. Transparent film: Receives toner ink.
3. Toner ink layer: Contains the printed image.
4. Transfer aid (Glue W1+): A specially formulated transfer aid designed to adhere the toner ink to the desired surface.

In this transfer method, the laser toner ink was printed on the transparent film and then transferred onto the desired surface using Sunnyscopa’s Glue W1+, which is specifically formulated for use with type-A film-free decals. This glue ensures optimal adhesion and is essential for the proper functioning of the transfer process on non-porous surfaces like ceramics, glass, and coated steel. After the transfer, the transparent film was removed, leaving only the toner ink image on the surface.

**Figure 4:**
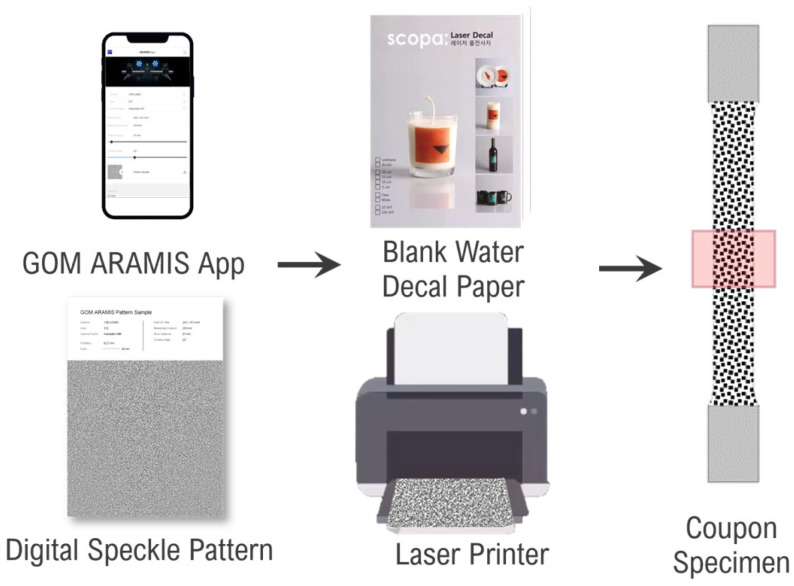
Process for generating and applying digital speckle pattern using Sunnyscopa film-free water decal paper.

**Figure 5:**
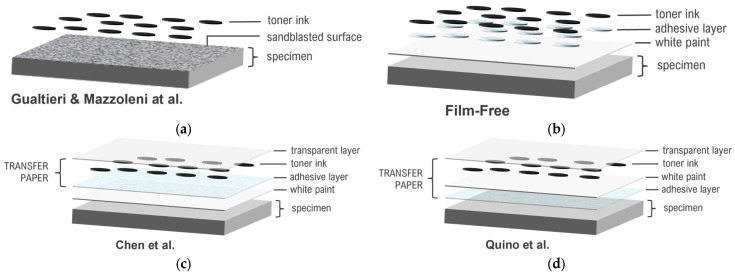
Cross-sectional comparison of speckle pattern application methods on a specimen: (**a**) Gualtieri and Mazzoleni et al.’s method, where toner ink is applied over a sandblasted surface [29,30]; (**b**) the film-free method, where only toner ink remains on the white paint after the transfer process, with no transparent layer left on the surface; (**c**) Chen et al.’s method, involving multiple layers, including a transparent layer, toner ink, and adhesive layer [31]; (**d**) Quino et al.’s method, similar to Chen’s, but with a slightly different assembly process [32].

Based on this principle, this study’s proposed method involves utilizing this water decal to transfer digital speckle patterns to metal surfaces. The process involved using the GOM ARAMIS App to generate the DSPs, printing the DSPs on blank film-free water decal paper using a laser printer, and applying the printed decals to cleaned specimen surfaces coated with white paint to achieve high contrast. This process is illustrated in Figure 4, and a cross section of the specimen (highlighted area) is illustrated in Figure 5b.

The advantage of this method is the control it provides over the design of the digital speckle patterns, which can be adjusted based on experimental needs. Using the GOM ARAMIS App, users can customize DIC parameters, such as field of view (FOV), camera angle (for 3D-DIC), and camera and lens types. This method ensures that the digital speckle patterns meet the suggested characteristics from the literature and International Digital Image Correlation Society (iDICs) guidelines [14,35,36,37,38,39], such as the 3–5 pixel-size requirement, and are limited only by the resolution of the printer and the specifications of the camera and lenses used. Figure 5 illustrates the cross-sectional layers of the speckle pattern application methods used in the studies by Gualtieri and Mazzoleni [29,30], Chen [31], Quino [32], and the film-free method. The comparison highlights the variations in layer compositions, from Gualtieri and Mazzoleni’s sandblasting approach to the multi-layered techniques employed by Chen and Quino, which incorporate transfer paper structures. The film-free method distinguishes itself by leaving only the toner ink on the white paint surface after the transfer process, with no residual transparent layer, simplifying the procedure while maintaining high-quality speckle patterns.

#### 3.1.2. Development and Optimization

To successfully apply the decal to metal surfaces, a series of preliminary tests was essential to optimize the procedure. These tests involved experimenting with different printer settings to identify the optimal combination for achieving the highest contrast and resolution of the toner ink. The choice of printer settings can significantly impact the quality of the speckle patterns. For example, higher-resolution settings ensure that the printed speckles are sharp and well-defined, reducing the likelihood of edge blurring or ink bleeding, which can occur with lower-quality settings or inappropriate paper types. Such factors are essential for maintaining the precision required for accurate DIC measurements. The printer used in this study was a Samsung C145x laser printer (Samsung, Suwon, Gyeonggi, Republic of Korea), configured with settings optimized for this application: Document Type set to “CAD”, Quality set to “High Image Quality”, and Paper Type selected as “Glossy Photo” with a weight of 176–220 g/m^2^.

Subsequently, preliminary applications on blank stainless-steel cards were performed to familiarize with the transfer process and evaluate the potential benefits of applying a top coat for added contrast without causing glare. Although not mandatory, applying a top coat to the transferred toner ink was deemed beneficial for our specific experimental needs, providing extra protection against scratches during transportation and handling, especially since the specimens needed to be facedown for strain gauge attachment. Based on our findings, a thin layer of Glue W1+, the same product used for the transfer, was determined to be the best option for top-coating.

**Figure 6:**
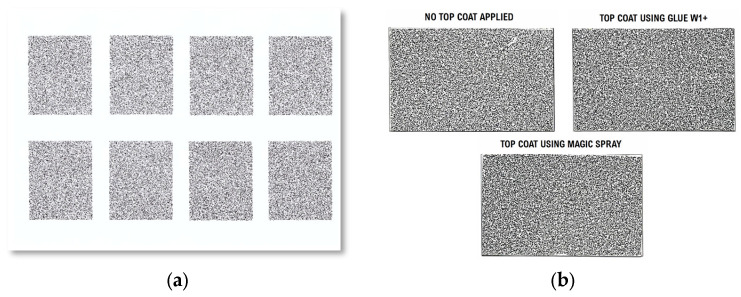
Optimization and preliminary testing for speckle pattern application: (**a**) print tests for optimizing printer settings; (**b**) evaluation of top coatings on stainless-steel cards to enhance speckle pattern quality.

Readers are encouraged to conduct their own testing to determine the most suitable printing parameters and top coatings based on their specific experimental conditions. These steps are not intended as strict guidelines, but rather to illustrate the thorough process undertaken to achieve optimal speckle pattern quality using the film-free method. Figure 6 shows the results of the print tests and top-coating evaluations conducted during the optimization process.

#### 3.1.3. Transfer Procedure on Specimens

After determining the optimal settings, the application procedure was validated using coupon specimens. Two point sizes of digital speckle patterns were used: 0.45 mm and 0.3 mm. This approach demonstrated the method’s capability to produce modifiable speckle patterns, with smaller point sizes enabling a higher spatial resolution or targeting smaller regions of interest (ROI). The GOM ARAMIS 2D system, which has been proven effective for capturing high-resolution images and performing detailed analyses in various DIC applications [40], was employed in this study. This system’s capabilities of providing precise deformation measurements and its successful use in previous studies underline its suitability for the current investigation. Table 1 summarizes the DIC measurement parameters for the two point sizes using the ARAMIS Adjustable 12M camera system, while Table 2 provides the technical specifications of the camera system [41].

**Figure 7:**
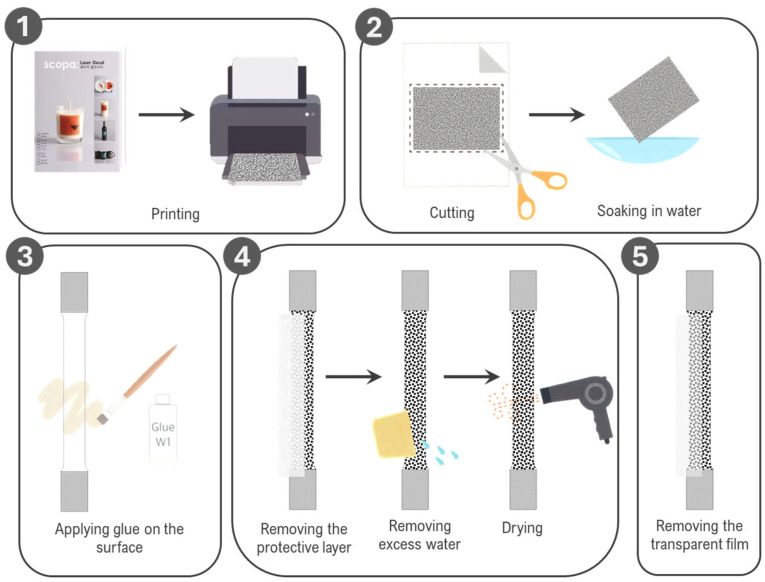
Step-by-step application of digital speckle pattern using Sunnyscopa film-free water decal paper.

The following steps outline the detailed process for applying the digital speckle patterns onto the specimens using the film-free water decal method. The specimens were coated with white paint first, before proceeding with the transfer. These steps are illustrated in Figure 7.

1. Printing: The DSPs, both 0.45 mm and 0.3 mm point sizes, were printed on the coated side of the film-free water decal sheets using the identified optimal printer settings.
2. Cutting and soaking: Each printed DSP was carefully cut to match the shape of the specimen, leaving a small margin around the edges. The cut DSPs were soaked in water for 3–5 s to activate the adhesive.
3. Gluing and application: Adhesive was evenly applied to the surface of each specimen using a brush. The soaked DSPs were then placed face down onto the specimen surface, ensuring proper alignment and coverage of the region of interest.
4. Protective layer removal and drying: The top paper layer of the water decal was gently removed, leaving only the transfer film in place. Excess adhesive, water, and air bubbles were carefully removed by gently rubbing the surface with a rubber scraper. The specimens were dried using a hair dryer for 5 min to securely bond the toner ink to the surface.
5. Transfer film removal: Once cooled, the transfer film was removed, leaving only the toner ink behind. To protect the toner, a thin layer of adhesive was applied over the surface and dried again using a hair dryer for an additional 3 min.

**Figure 8:**
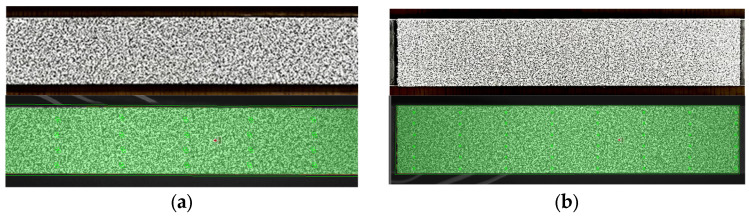
Quality assessment of digital speckle patterns applied on SS275 steel specimens: (**a**) 0.45 mm point size; (**b**) 0.3 mm point size.

The film-free method was applied to SS275 steel specimens. Reference images for each point size type were captured using GOM Snap 2D 2019. These images were then analyzed using GOM Correlate 2019. The surface component was created using the settings described in Table 3. For the 0.45 mm specimens, square subsets of 19 × 19 pixels with a step size of 16 pixels were used. For the 0.3 mm specimens, 15 × 15 pixels subsets with a step size of 7 pixels were used. The facet overlaps and other DIC parameters presented in Table 3 were selected based on a combination of recommendations from the ARAMIS device manufacturer, training received from their expert technician, guidelines provided by the iDICs, and the specific requirements of this study. The software assessed the stochastic pattern quality, classifying it using a color-coded system: red indicating poor computation quality (insufficient for creating a surface component), yellow indicating moderate quality (requires improvement), and green indicating good computation quality. Both GOM Snap 2D and GOM Correlate used in this study are part of GOM Software 2019, with version 2019 Hotfix 7, Rev. 128764, Build 2020-06-18. Figure 8 shows the specimens after the application of the speckle patterns and their quality assessment.

The figure illustrates that both point sizes produce uniform green areas, signifying good stochastic patterns. Notably, the 0.3 mm point size detected more points, indicating a higher spatial resolution. This highlights a significant advantage of the film-free water decal method. The adjustability of the Digital Speckle Pattern (DSP) allows for customization and modification to meet the specific requirements of the experiment. This flexibility enables the generation of higher resolution DIC images, leading to more precise measurements. Furthermore, the ability to achieve such a high resolution with the film-free water decal method is particularly advantageous for experiments that necessitate detailed strain distribution analysis.

### 3.2. Experimental Validation

#### 3.2.1. Experiment Design

While consistent speckle patterns with different speckle sizes have been produced, it is essential to determine whether they perform well in DIC experiments. The primary objective of the experimental validation is to assess whether the film-free water decal method can serve as a viable alternative to traditional spray-painting. This method aims to eliminate the variability in speckle patterns that is often encountered with spray painting. This experiment compares the effectiveness of the film-free water decal method against traditional spray painting in terms of speckle pattern quality, measurement accuracy in strain, and material property values obtained.

**Figure 9:**
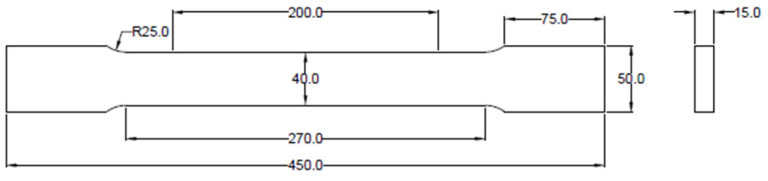
Geometry of tensile coupon specimen (units in mm).

Two specimens were prepared: one with a speckle pattern applied using the film-free water decal with a 0.45 mm point size, and another using the traditional spray-painting method. Both specimens were made of SS275 structural steel coupons. The dimensions of the coupons adhered to ASTM E8/E8M—22 Standard Test Methods for Tension Testing of Metallic Materials [42], utilizing plate-type specimens. Additionally, the chemical composition and material properties of the SS275 steel are in accordance with the KS D 3503 standard. The specific dimensions are shown in Figure 9.

Since the specimens were plate-type, 2D-DIC was used in this experiment as it is suitable for planar specimens. Both specimens were subjected to tensile loading. The loading speed for the tensile test was controlled using the Crosshead Speed Control Method specified in ASTM E8/E8M—22, which is used for determining yield properties. According to this method, the testing machine was set to a crosshead speed equivalent to 0.015 mm/mm/min of the original reduced parallel section. Given that the specimens had a 270 mm reduced parallel section, the crosshead speed was set to 4.05 mm/min.

**Figure 10:**
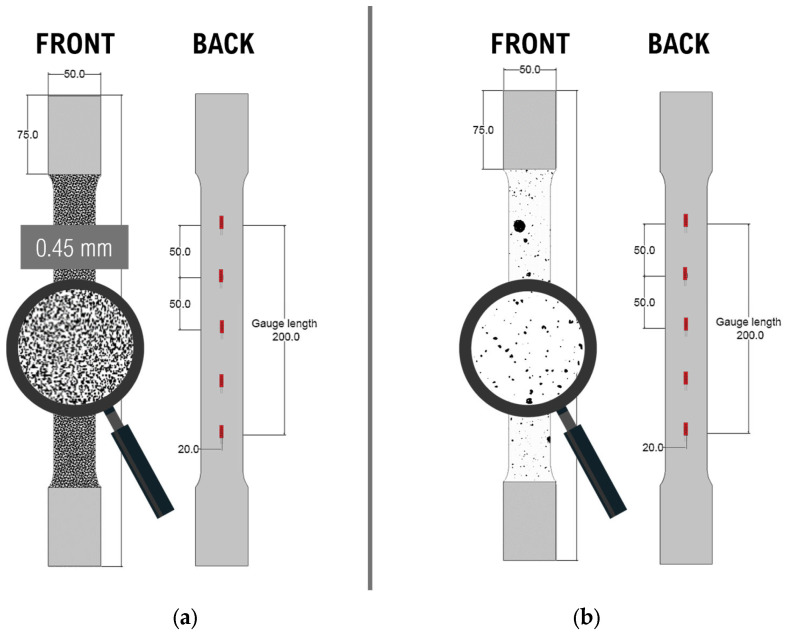
Schematic representations of film-free and spray-painted specimens showing speckle patterns and strain gauge locations (units in mm): (**a**) film-free specimen; (**b**) spray-painted specimen.

To assess the ability of the film-free method in obtaining strain values for local strain analysis, the strains measured by DIC will be compared to those measured by strain gauges. Strain gauges were attached at specific locations on the back of each specimen to facilitate comparison of the DIC results between spray-painted and film-free specimens. Figure 10 shows a schematic representation of the front and back views of both types of specimens. The front view highlights the region with the speckle patterns applied, while the back view shows the locations for strain gauges. Each specimen had 5 strain gauges attached along the center line of the gauge length, spaced equally at 50 mm intervals. FLAB-5 foil strain gauges, manufactured by TML Japan, were used. These gauges are designed as single-axis sensors suitable for general-purpose strain measurement applications. The strain gauges serve as a benchmark for comparing the two methods.

**Figure 11:**
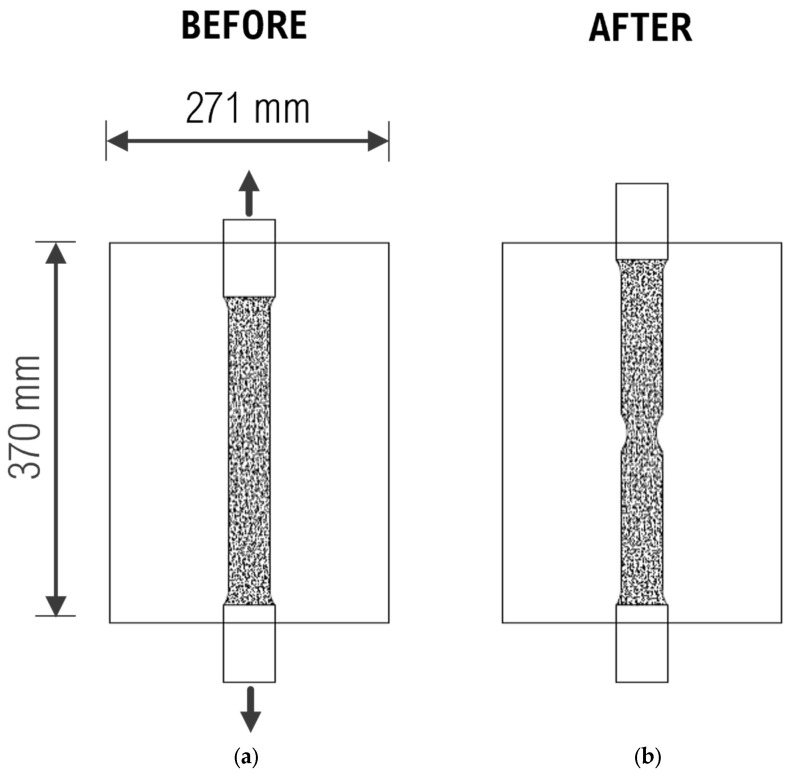
Field of view for DIC analysis: (**a**) before loading; (**b**) after loading.

The quantities of interest for this experiment were strain for local strain analysis and displacements for characterizing the materials for stress–strain curves. Given that this was a tensile test, it was important to measure the elongation of the gauge length section of the coupon specimens. Therefore, the region of interest was the area along the gauge length. As this region of interest was within the field of view used in the DIC parameters listed in Table 1 for the 0.45 mm point size, these parameters were used in the experiment. Figure 11 shows the field of view using these DIC parameters, depicting the specimen both before and after loading, along with the expected elongation of the material.

#### 3.2.2. Specimen Preparation

**Figure 12:**
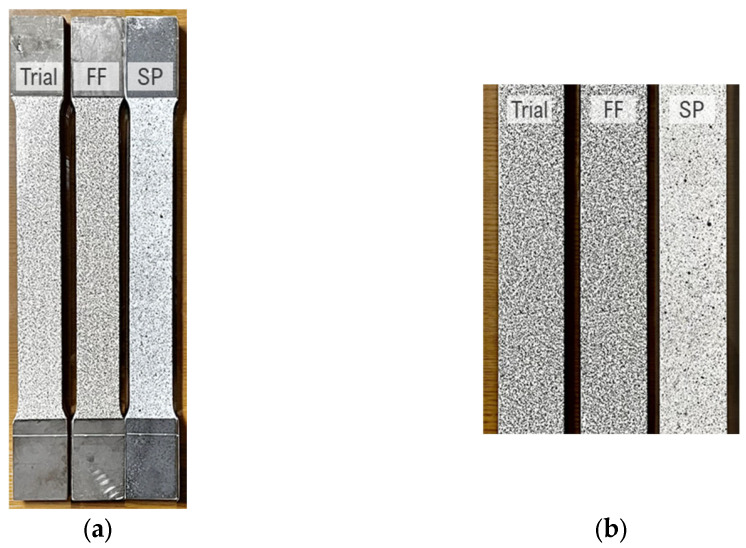
Application of speckle pattern on specimens: (**a**) specimens with applied speckle patterns: trial, film-free (FF), and spray paint (SP); (**b**) close-up view of speckle patterns.

Before the speckle pattern application, the specimen surfaces were meticulously cleaned to remove any contaminants, such as oil and dirt, which could interfere with the adhesion of the speckle patterns. Both types of specimens were then coated with white paint. For the film-free specimens, the procedure outlined in Section 3.1.3 was followed. For the spray-painted specimens, Dupli-color Aqua ECO+ spray paint was used, with “Pure White” for the base layer and “Deep Black” for the speckle patterns. The application of speckle patterns on the specimens is illustrated in Figure 12.

**Figure 13:**
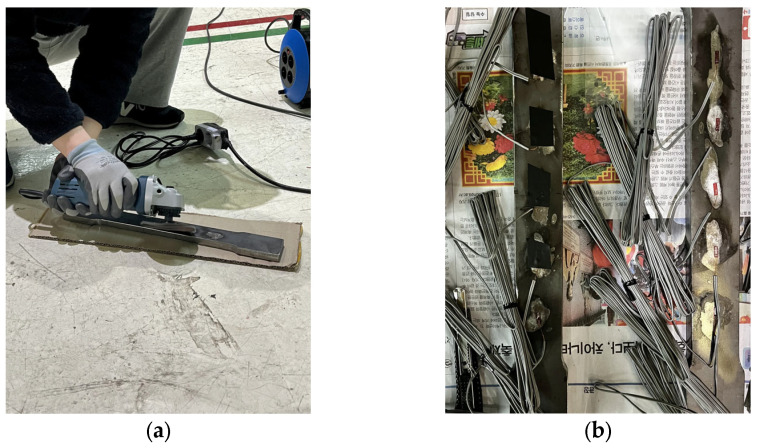
Preparation of specimens for strain gauge attachment: (**a**) grinding the back of a specimen for strain gauge attachment; (**b**) specimens with attached strain gauges.

The locations for the strain gauges at the back of the specimens were ground to remove the coating of the steel, ensuring the proper adhesion of the strain gauges to the surface. After grinding, the specimens were cleaned with alcohol to remove any dust from grinding and grease residues. Subsequently, the strain gauges were carefully attached to the marked positions, and coating tape was applied to secure the strain gauges during the experiment. Figure 13a illustrates the process of grinding the surface to prepare for strain gauge attachment. Figure 13b shows the specimens with the attached strain gauges.

#### 3.2.3. Experimental Setup

**Figure 14:**
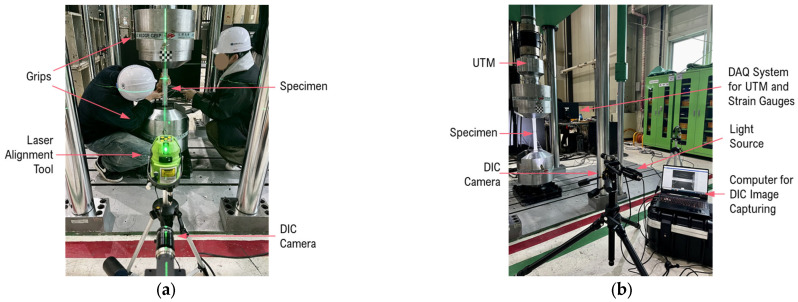
Alignment and complete test setup for DIC and strain gauge measurements: (**a**) alignment of the DIC camera, and specimen with the grips using a laser alignment tool; (**b**) overview of the complete test setup, including the DIC system and DAQ system.

The experiment was conducted at the Seismic Research and Test Center (SESTEC) at Pusan National University. The primary machine used for tensile testing was a Universal Testing Machine (UTM) with a 1000 kN capacity. The specimens were securely gripped in the testing machine. The camera was positioned to meet the required measuring distance for achieving the desired field of view. A laser distance measure was employed to verify the distance accuracy. Additionally, a laser alignment tool was used to ensure the proper alignment of the specimen with the grips of the UTM and the camera. Figure 14a illustrates the alignment and setup of the experiment, showing how the camera and specimen were positioned to achieve accurate measurements. Figure 14b provides an overview of the test setup, with the DIC system and the DAQ system.

#### 3.2.4. DIC Setup and Processing

In preparation for DIC analysis, a systematic setup procedure was implemented to optimize hardware and software settings for capturing high-quality images of specimen deformation. This procedure involved precise adjustments in the lighting, camera settings, and exposure parameters to ensure clear and static images during mechanical testing. GOM Snap 2D 2019 was utilized to capture images in real time during the tensile testing process. The following steps outline the detailed setup process for DIC image acquisition:

1. Position and adjust lighting: The lighting was carefully positioned and adjusted to evenly illuminate the region of interest.
2. Set aperture: The camera aperture was set to the maximum (lowest f-stop value) to achieve the shortest possible exposure time without compromising the resolution.
3. Optimize exposure time: A low exposure time was selected to minimize motion blur during specimen stretching and tensile loading, ensuring static images.
4. Verify exposure values: Exposure values were verified to ensure they were below the calculated minimums.
5. Adjust filters: Filters were adjusted to eliminate glare and enhance image clarity.

According to the iDICs guidelines [14], there are recommended tips for determining the appropriate frame rate and exposure time. The guide suggests a maximum displacement between frames of approximately one subset size. For postprocessing, a subset size of 19 × 19 pixels was used. A frame rate of 3 Hz was selected, which exceeds the minimum required frame rate of 0.0394 Hz, considering the image scale and test speed of 4.05 mm/min. This equates to capturing 3 images per second throughout the test’s duration.

Determining the appropriate exposure time is crucial to limit motion blur and ensure clear, static images for DIC analysis. According to the iDICs guidelines, a conservative estimate for the maximum allowable test piece motion is approximately 0.01 pixels over time in typical DIC setups. The displacement per exposure in pixels is calculated using the following formula:

Displacement [px] = (Velocity [mm/s]) × (Image Scale [px/mm]) × (Exposure Time [s])(1)

The maximum exposure time was calculated to be 13.3 ms, ensuring that these values remained well below the limit set by the inverse of the frame rate, which is 330 ms. This effectively minimizes motion blur, providing clear and static images essential for accurate DIC analysis.

All these hardware and software variables, combined with the field of view and measuring distance, significantly impact the overall quality of DIC image outcomes, which is critical for DIC processing. Pattern quality was assessed before testing to ensure optimal image processing by the software.

Before commencing formal testing, a trial test was conducted to assess the adequacy of DIC settings in capturing clear images without motion blur. This trial also evaluated whether the selected 3 Hz frame rate was sufficient and if the image measuring sequence in the software could accommodate the entire test duration.

After obtaining the images, GOM Correlate 2019 was employed for postprocessing and analysis, enabling the extraction of displacement and strain data for comprehensive deformation analysis. A surface component was created for each specimen to facilitate DIC calculations. Each surface component utilized a subset size of 19 × 19 pixels, ensuring uniformity and consistency in the analysis across all tests. Detailed parameters employed for DIC analysis are summarized in Table 4.

## 4. Results

### 4.1. Pattern Quality Assessment

**Figure 15:**
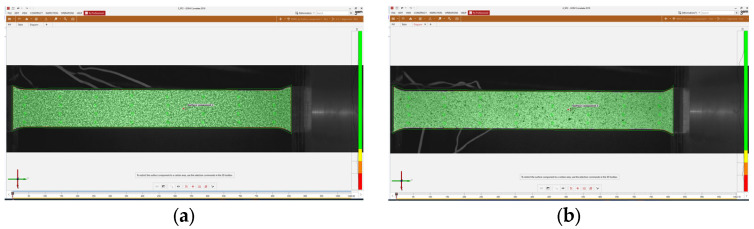
Pattern quality assessment of speckle patterns for DIC analysis: (**a**) film-free method; (**b**) spray-painted method.

Before initiating the tensile tests, a reference image was captured to assess the pattern quality. The detected point values exhibit consistency across specimens. Images of the pattern quality checks for film-free and spray-painted methods are shown in Figure 15. A total of 6534 points was detected for the film-free method, and 6479 points were detected for the spray-painted method. This consistency of detected points across different specimens suggests that both methods are capable of generating high-quality speckle patterns suitable for DIC analysis.

**Figure 16:**
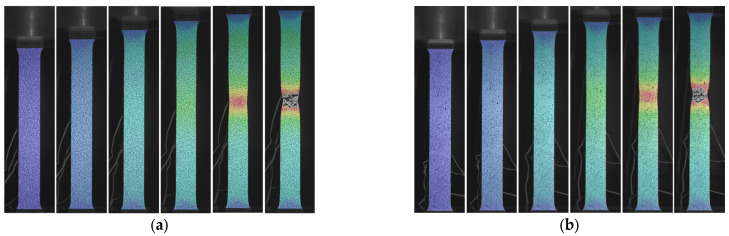
Strain distribution at different stages of tensile testing: (**a**) film-free method; (**b**) spray-painted method.

Additionally, the performance of the speckle pattern was evaluated throughout the duration of the tensile testing. Figure 16 illustrates the strain distribution at different stages of tensile testing for both methods. It can be observed that accurate strain calculations were maintained up to the point of fracture for both the film-free and spray-painted methods. This indicates that both methods provided reliable data throughout the entire testing process, demonstrating their effectiveness in capturing strain distribution during tensile loading.

### 4.2. Validation of Strain Measurements

**Figure 17:**
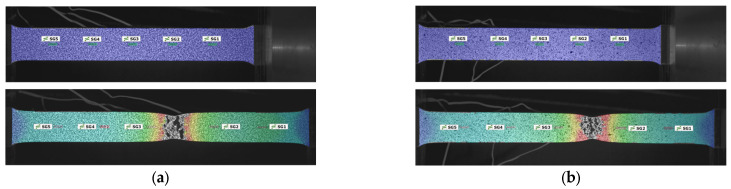
Virtual strain gauges created in GOM Correlate showing images before and after loading: (**a**) film-free specimen; (**b**) spray-painted specimen.

**Figure 18:**
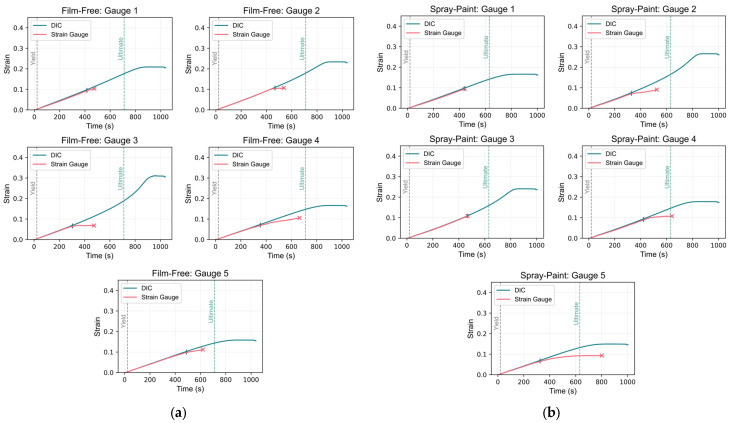
Comparison of strain data from DIC and actual strain gauges: (**a**) film-free specimen; (**b**) spray-painted specimen.

Virtual strain gauges were created in GOM Correlate by defining a component region measuring 10 mm × 3 mm and obtaining the average strain within this area at the corresponding locations of the physical strain gauges on the undeformed images. Figure 17 presents images before and after loading (reference and after fracture, respectively), highlighting the virtual strain gauges. Subsequently, strain data from both the actual strain gauges and the DIC virtual strain gauges were plotted for each location, as shown in Figure 18.

Upon examining the graphs, it is evident that the actual strain gauge readings conclude prematurely, indicated by “x” markers for both specimens. In contrast, the DIC readings persist until the conclusion of loading, including the fracture point. For the film-free specimen, the image post-failure shows that the break occurred between strain gauge locations 3 and 2, with the highest strain magnitude observed near gauge location 3. This is captured by the DIC measurements, as the plots illustrate that the DIC strain values peak at gauge 3, followed by gauge 2. However, the actual strain gauge readings do not reflect this trend due to their premature termination, which may have been caused by gauge overload during the experiment.

Similarly, for the spray-painted specimen, cracking occurred between strain gauge locations 2 and 3, closer to gauge 2. In the plots comparing the DIC-derived and actual strain values, the highest strain values are observed at strain gauge location 2, followed by location 3. This aligns with the fracture location observed in the DIC measurements between these two points. The lowest strain values are at strain gauge location 5, which is furthest from the fracture site. This distribution of strain values indicates that the strain concentration was highest near the crack, as expected since material deformation is most significant in this region.

Subsequently, the strain readings at the yield point for both DIC and actual measurements were compared. Comparing strain values at a specific point, such as the yield point, provides a direct assessment of the agreement between DIC-derived strain measurements and actual strain gauge measurements under critical loading conditions. The yield point is critical as it marks the transition from elastic to plastic deformation, significantly impacting material performance. The differences between DIC and actual strain gauge measurements at this point were calculated and summarized in Table 5. The observed differences were minimal, typically reaching only the fourth decimal place.

However, comparing strain values at a single point does not fully capture the similarity between readings throughout the entire loading process. To address this, similarity measures used in time-series analysis were computed, including the Pearson correlation coefficient and Euclidean distance. These were employed for this analysis until the end of the actual strain gauge readings (“x”).

The Pearson correlation coefficient assesses the strength and direction of the linear relationship between datasets. A value close to 1 indicates a strong positive linear relationship, implying a high correlation between the DIC-derived and actual strain measurements. Conversely, a value near −1 suggests a strong negative linear relationship, while a value near 0 indicates no linear relationship. Euclidean distance measures the geometric difference between datasets by calculating the straight-line distance between corresponding points. A smaller Euclidean distance signifies a closer agreement between the datasets in both magnitude and direction. These measures provide a comprehensive perspective on the overall agreement between DIC-derived and actual strain measurements across multiple data points. Additionally, the time until the difference between DIC and actual strain readings falls below a threshold of 0.005 was determined, offering insights into the duration of their alignment. This time point is denoted by a vertical line (“|”) on the plots. The similarity measures are presented in Table 6.

The high Pearson correlation coefficients for each gauge location imply a strong positive linear relationship between the DIC-derived and actual strain measurements. This suggests a high degree of correlation between the two datasets, affirming the reliability of the DIC-derived strain values compared to the actual strain gauge readings until just before the termination of the actual strain gauge readings. Moreover, the small Euclidean distances between the DIC-derived and actual strain measurements further support the notion of close agreement and similarity between the datasets. The small differences in magnitude and direction between corresponding points in the two datasets suggest minimal geometric discrepancies or errors, which could be attributed to either the DIC-derived strain values or the strain gauge measurements due to the potential degradation or early failure of the strain gauges.

**Figure 19:**
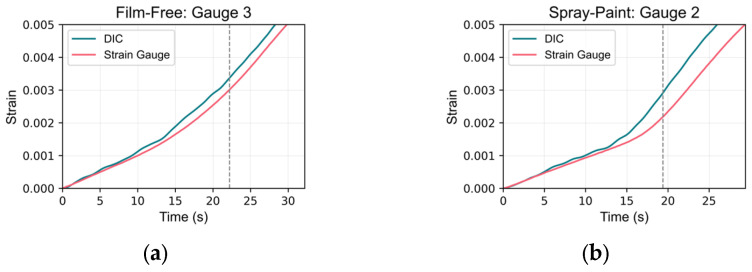
High-error zoomed-in plots at the yield point: (**a**) film-free specimen: gauge 3; (**b**) spray-painted specimen: gauge 2.

The zoomed-in plots for the highest errors at the yield point are shown in Figure 19 for:

- Film-free specimen at strain gauge location 3; and
- Spray-painted specimen at strain gauge location 2.

These plots reveal similar trends in the strain data; however, discrepancies in the strain values at the yield point are evident, likely resulting from differences in the onset of strain increase. This suggests potential synchronization issues. The start of the readings for the DIC virtual strain gauges was aligned based on the time when displacement values in the virtual extensometer began to increase.

The observed errors likely stemmed from asynchronous start times between the DIC system and the UTM. Specifically, the DIC image capturing started before the UTM loading and sensor data recording, causing initial alignment issues. Adjusting the start times of the datasets reduced the error, highlighting the critical impact of synchronization on measurement accuracy. This limitation arose because the current DIC system was not integrated with the UTM to read force values simultaneously. Future improvements could involve using synchronized equipment to ensure concurrent data acquisition, thereby reducing these errors.

Overall, the comparison between film-free and spray-painted specimens demonstrates a high degree of agreement between DIC-derived strain measurements and those obtained from actual strain gauges. This consistent alignment indicates that both speckle pattern application methods—film-free water decal and traditional spray-painting—are effective in capturing the mechanical deformation behavior of the specimens. The results also affirm that DIC, in general, is a reliable, non-contact, and non-destructive method for strain measurement and analysis in comparison to strain gauges. Although general-use strain gauges were used in this study, future studies could benefit from using high-performance strain gauges for an improved comparison with DIC. These findings suggest that the proposed film-free water decal method can serve as a viable alternative to the traditional spray-painting technique, potentially offering advantages in terms of application ease and pattern consistency.

### 4.3. Evaluation of Material Properties

**Figure 20:**
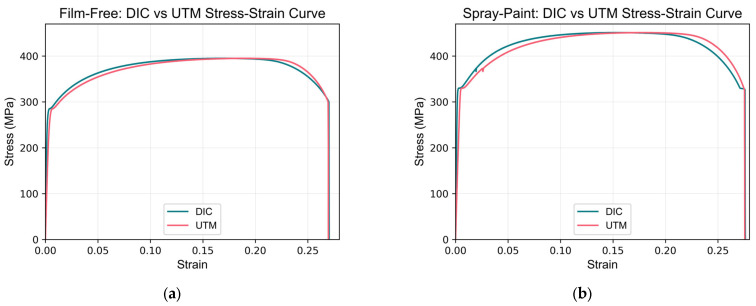
Stress–strain curves comparing DIC and UTM measurements: (**a**) film-free specimen; (**b**) spray-painted specimen.

A detailed comparison of the material properties derived from both DIC and UTM data was conducted. The properties analyzed include Young’s modulus (E), yield strength (using both extension-under-load (EUL) and offset methods) and their corresponding strains, ultimate tensile strength (UTS) and its strain, fracture stress and strain, and toughness. The stress–strain curves for both the film-free and spray-painted specimens obtained from DIC and UTM measurements are presented in Figure 20 and summarized in Table 7.

The results show minor discrepancies between the UTM- and DIC-derived material properties. The percentage differences in Young’s modulus, yield strength, and toughness are relatively small, indicating a high degree of correlation between the two methods. Some discrepancies in certain specimens can be attributed to inherent differences in data acquisition methods and the use of UTM data instead of an external extensometer gripped at the gauge length marks. This study did not use a separate extensometer due to its potential to obstruct the DIC speckle patterns, which could affect the accuracy of the strain measurements.

Despite these differences, the strong agreement between UTM- and DIC-derived values supports the reliability of DIC as a method for material characterization. The comparison between film-free and spray-painted methods revealed minimal errors for both techniques, indicating that the film-free method can be a viable alternative to traditional spray painting. The small percentage differences observed in the material properties measured using both methods further affirm the effectiveness and reliability of the film-free water decal method. This suggests that the film-free method offers comparable accuracy to spray-painting while potentially providing advantages in terms of application ease and pattern consistency.

## 5. Conclusions

This study demonstrated the effectiveness and reliability of the film-free water decal method as a viable alternative for speckle pattern application in Digital Image Correlation (DIC). The comparative analysis with traditional spray-painting techniques revealed minimal errors in strain and displacement measurements, validating the high degree of correlation between the two methods. The film-free method offers significant advantages, including superior control over speckle pattern application, improved pattern consistency, and ease of use. It enables the generation of high-quality, high-contrast speckle patterns without extensive surface preparation or specialized equipment. Additionally, the method’s adaptability allows for higher resolution in DIC images, making it particularly beneficial for experiments requiring detailed strain distribution analysis. The strong agreement between DIC-derived values and those obtained from traditional methods supports the film-free water decal method as a practical and reliable tool for material characterization across various engineering and scientific fields.

## Figures and Tables

**Table 1:** DIC measurement parameters for different point sizes.

DIC Measurement Parameters	Point Size	
	0.45 mm	0.3 mm
Field of View	370 × 271 mm^2^	245 × 180 mm^2^
Measuring Distance	1409 mm	967 mm
Local Resolution	11.1 pixel/mm	16.7 pixel/mm
Sample Pattern	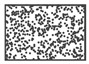	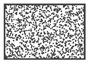

**Table 2:** Technical specifications of the ARAMIS Adjustable 12M camera system.

Feature	Specification
Camera Resolution	12M (4096 × 3000) pixels
Pixel Size	3.45 µm
Frame Rate	25 fps (full frame) to 150 fps (1/6 image)
Lens Focal Length	50 mm

**Table 3:** DIC processing parameters for different point sizes.

DIC Processing Parameters	Point Size	
	0.45 mm	0.3 mm
Subset Size *	19 × 19 pixels	15 × 15 pixels
Step Size *	16 pixels	7 pixels
Computation Method	More points	More points
Number of Points Detected	6534	43,735

* In GOM Correlate, “Subset” is referred to as “Facet” and “Step Size” as “Point Distance”.

**Table 4:** DIC processing parameters.

DIC Parameters	Point Size
Subset Size *	19 × 19 pixels
Step Size *	16 pixels
Computation Method	More points
Facet Matching	Against previous stage
Start Facets	Automatic
Track to Component	No
Strain Tensor Neighborhood	1
Interpolation Size	2

* In GOM Correlate, “Subset” is referred to as “Facet” and “Step Size” as “Point Distance”.

**Table 5:** Strain values at the yield point for DIC and strain gauge measurements.

		Gauge 1	Gauge 2	Gauge 3	Gauge 4	Gauge 5
Film-Free Method	DIC	0.003324	0.003394	0.003367	0.003109	0.003010
	Strain Gauge	0.003019	0.003058	0.003011	0.002994	0.003024
	Abs. Difference	0.000305	0.000336	0.000356	0.000115	0.000013
	Percent Error	10.12%	10.97%	11.83%	3.84%	0.44%
Spray-Paint Method	DIC	0.002632	0.002917	0.002563	0.002413	0.002598
	Strain Gauge	0.002463	0.002186	0.002201	0.00217	0.002245
	Abs. Difference	0.000169	0.000731	0.000361	0.000243	0.000353
	Percent Error	6.87%	33.45%	16.40%	11.22%	15.72%

**Table 6:** Similarity measures for DIC and strain gauge measurements.

		Gauge 1	Gauge 2	Gauge 3	Gauge 4	Gauge 5
Film-Free Specimen	Consistency Duration (s)	415.6	468.0	303.9	353.7	488.8
	Pearson Coefficient	0.999850	0.994194	0.954884	0.990494	0.997355
	Euclidean Distance	0.000305	0.000336	0.000356	0.000115	−0.000013
Spray-Painted Specimen	Consistency Duration (s)	440.4	328.3	467.1	421.9	326.0
	Pearson Coefficient	0.999934	0.979428	0.99997	0.986352	0.966609
	Euclidean Distance	0.000169	0.000731	0.000361	0.000243	0.000353

**Table 7:** Material properties comparison for DIC and UTM measurements.

	Film-Free Specimen			Spray-Paint Specimen		
Material Properties	DIC	UTM	Percent Error	DIC	UTM	Percent Error
E [MPa]	215,370.1	208,018.9	3.53%	232,801	206,446.2	12.77%
Yield Strength (EUL) [MPa]	286.0613	295.4122	3.17%	330.7935	340.4882	2.85%
Yield Point (EUL) Strain	0.00500	0.00501	0.24%	0.00500	0.00501	0.06%
Yield Strength (Offset) [MPa]	283.7237	286.3742	0.93%	330.0682	332.3192	0.68%
Yield Point (Offset) Strain	0.00332	0.00338	1.76%	0.00342	0.00361	5.32%
Ult. Tensile Strength (UTS) [MPa]	395.0626		-	451.2659		-
Ult. Tensile Strain	0.16749	0.18626	10.08%	0.15192	0.17473	13.05%
Fracture Stress [MPa]	297.7575		-	325.4922		-
Fracture Strain	0.27009	0.26956	0.20%	0.27581	0.27536	0.17%
Toughness [MPa]	100.10664	99.11616	1.00%	116.45652	115.68712	0.67%

## Data Availability

The data that support the findings of this study are included in the article. Additional raw data are available from A.C.S., upon reasonable request.
